# YAP1 Is Associated with Cumulus Expansion-Related Factor Expression and Autophagy-Related Signaling in Yak Cumulus Cells

**DOI:** 10.3390/ani16172636

**Published:** 2026-08-22

**Authors:** Tiantian Zhang, Meng Wang, Xin Ma, Tingting Lu, Qiyong Zuo, Qian Zhang, Libin Wang, Yangyang Pan

**Affiliations:** 1 College of Veterinary Medicine, Gansu Agricultural University, Lanzhou 730000, China; 1073325020253@st.gsau.edu.cn (T.Z.); wangmeng@gsau.edu.cn (M.W.); mxinmaxin@163.com (X.M.); 18693297203@163.com (T.L.); zuoqy@st.gsau.edu.cn (Q.Z.); zq880204@126.com (Q.Z.); wanglb@gsau.edu.cn (L.W.); 2 Gansu Livestock Embryo Engineering Technology Innovation Center, Lanzhou 730070, China

**Keywords:** autophagy, cumulus expansion, Hippo signaling, yak, Yes-associated protein 1

## Abstract

Cumulus expansion is important for oocyte maturation, but its molecular regulation in yak remains unclear. This study examined the association of Yes-associated protein 1 (YAP1) with cumulus expansion-related and autophagy-related molecular markers in cumulus cells isolated from yak cumulus–oocyte complexes collected from ovarian follicles. YAP1 was overexpressed or knocked down, and YAP1-overexpressing cultures were additionally treated with 3-methyladenine (3-MA). Transcript abundance was assessed by RT-qPCR and interpreted descriptively, whereas protein abundance was evaluated by Western blotting; representative immunofluorescence and mCherry-GFP-LC3B images were also examined. YAP1 overexpression significantly increased HAS2 and PTGS2 protein abundance, while PTX3 and TNFAIP6 showed non-significant upward trends; YAP1 knockdown significantly reduced all four proteins. YAP1 manipulation was also associated with changes in Beclin-1, ATG5, p62, and the LC3B-II/LC3B-I ratio. Compared with YAP1 overexpression alone, 3-MA co-treatment significantly reduced HAS2, PTGS2, PTX3, TNFAIP6, Beclin-1, and ATG5, increased p62, and numerically reduced the LC3B-II/LC3B-I ratio without statistical significance. These findings support an association between YAP1 and cumulus expansion-related molecular programs and are consistent with partial involvement of 3-MA-sensitive, autophagy-associated signaling. Direct functional assays and pathway-specific validation are required to establish causality.

## Simple Summary

Cumulus cells surround the oocyte and support its maturation. Their expansion before ovulation depends on coordinated changes in extracellular matrix-related molecules. This study examined whether YAP1, a regulator of cell growth and signaling, is associated with these molecular changes in cumulus cells isolated from yak cumulus–oocyte complexes collected from ovarian follicles. YAP1 was experimentally overexpressed in one set of cultures and knocked down in another. In a separate overexpression experiment, cultures were additionally treated with 3-methyladenine (3-MA), a pharmacological compound that affects signaling pathways involved in cellular recycling. YAP1 overexpression was associated with higher abundance of several cumulus expansion-related proteins and changes in autophagy-related markers, whereas YAP1 knockdown produced an overall opposite pattern. 3-MA co-treatment attenuated several molecular changes associated with YAP1 overexpression. These findings support an association between YAP1 and cumulus-cell molecular programs and are consistent with partial involvement of autophagy-associated processes. Because the study measured molecular markers rather than actual cumulus expansion or oocyte developmental outcomes, further functional studies are needed.

## 1. Introduction

Yak (*Bos grunniens*) is an important indigenous livestock species of the Qinghai–Tibet Plateau and its adjacent alpine regions, where its meat, milk, fiber, and draught value are of major importance to pastoral production and livelihoods. The restricted breeding season, long reproductive cycle, and limited multiplication rate of elite germplasm constrain yak reproductive efficiency and remain insufficient to meet the demands of genetic improvement and large-scale production. Assisted reproductive technologies, including in vitro fertilization and embryo transfer, provide effective approaches for accelerating the utilization of superior yak germplasm, and the acquisition of mature oocytes with high developmental competence is fundamental to the stable operation of in vitro embryo production systems. In recent years, molecular studies of yak oocyte maturation have progressively identified regulatory factors, including *GPR50*, HIF-1α, and *YAP1*/TAZ, as participants in meiotic resumption, cumulus-cell function, and subsequent embryonic development [1,2,3]. Nevertheless, the in vitro maturation rate and embryonic developmental quality of yak oocytes remain susceptible to culture conditions, and changes in the follicular microenvironment and the underlying cellular regulatory mechanisms require further clarification.

The cumulus–oocyte complex is the basic functional unit through which the oocyte acquires nuclear and cytoplasmic maturation competence. Through gap-junctional communication and paracrine signaling, cumulus cells supply the oocyte with pyruvate, amino acids, and reducing metabolites and participate in the maintenance and resumption of meiotic arrest; oocyte-secreted factors, in turn, regulate the responsiveness of cumulus cells to FSH and maturation stimuli [4,5]. Following the onset of maturation, cumulus cells synthesize and remodel a hyaluronan-rich extracellular matrix. Molecules including HAS2, PTGS2, PTX3, and TNFAIP6 act coordinately in this process, and their expression is commonly used to evaluate cumulus expansion and the quality of oocyte maturation. Recent studies in bovine oocytes have shown that redox status, IGF-1 bioavailability, and metabolic stress can alter cumulus-cell function and oocyte developmental competence. PAPP-A can enhance the antioxidant effect of IGF-1 in bovine in vitro embryo production systems; L-kynurenine can improve oxidative homeostasis in bovine oocytes, whereas elevated β-hydroxybutyrate can induce endoplasmic reticulum stress in bovine cumulus cells [6,7,8]. These findings indicate that cumulus cells are not merely a structural layer surrounding the oocyte but also an important cell population that senses and integrates the metabolic environment of the follicle. Cumulus cells coordinate nutrient transfer, redox homeostasis, gap-junctional communication, and extracellular matrix formation, thereby supporting oocyte growth, meiotic maturation, ovulation, and fertilization. During cumulus expansion, hyaluronan synthesized mainly by HAS2 interacts with matrix-associated proteins, including TNFAIP6 and PTX3, to form a stable extracellular matrix surrounding the oocyte [9,10].

Autophagy is an important homeostatic mechanism through which cells use lysosomes to degrade damaged organelles and abnormal macromolecules and maintain energy and material recycling. During oocyte maturation, cumulus cells must simultaneously undergo metabolic reprogramming, hormone secretion, and extracellular matrix synthesis; therefore, their autophagic status may directly affect their capacity to support the oocyte. In recent years, studies in yak and cattle have gradually revealed the role of autophagy in female reproductive cells of ruminants. Exosomes derived from yak follicular fluid can regulate estrogen metabolism by activating autophagy in cumulus cells, whereas EGF affects autophagic and endocytic activity in yak cumulus cells in a concentration-dependent manner [11,12]. HIF-1α-mediated regulation of autophagy is closely associated with yak cumulus expansion, oocyte maturation, and early embryonic development [2], and an appropriate concentration of rapamycin can improve the developmental competence of yak oocytes [13]. In addition, a 2025 bovine study showed that Mogroside III improved in vitro oocyte maturation and subsequent development by regulating autophagy in cumulus cells [14]. These findings suggest that autophagy may be an important cellular process linking the follicular microenvironment, cumulus-cell status, and oocyte quality, although its upstream regulatory network remains incompletely understood.

YAP1 and TAZ are the principal downstream transcriptional co-activators of the Hippo pathway, and their activity is regulated by phosphorylation status and subcellular localization. When the Hippo kinase cascade is suppressed, unphosphorylated YAP1/TAZ can enter the nucleus and cooperate with TEAD family transcription factors to regulate genes associated with cell proliferation, differentiation, metabolism, and the extracellular matrix [15]. In the bovine ovary, YAP1 participates in granulosa-cell proliferation and steroidogenesis [16]. Evidence from mouse genetic models further supports the importance of YAP1 in follicular somatic cells. YAP1 is dynamically expressed and activated during granulosa-cell development, whereas pharmacological inhibition or conditional deletion of Yap1 in proliferating granulosa cells disrupts follicular development, increases granulosa-cell apoptosis, and reduces female fertility [17]. These findings indicate that both YAP1 abundance and activation status are important for normal follicular-cell function, although observations obtained in mouse granulosa cells cannot be directly extrapolated to yak cumulus cells. FSH can regulate YAP-TEAD transcriptional activity in bovine granulosa cells, and YAP signaling also participates in the preovulatory EGF-like cascade [18,19]. In bovine cumulus cells, YAP/TAZ interacts with EGFR signaling and influences expansion-related events [20]. A recent study in yak further showed that activation of YAP1 and TAZ affects oocyte maturation, cumulus expansion-related molecules, oocyte-secreted factors, and blastocyst quality [1]. However, direct evidence linking YAP1 to expansion-related molecular changes and autophagy-associated processes in yak cumulus cells remains limited. Therefore, the present study used in vitro-cultured yak cumulus cells to examine autophagy-related markers and cumulus expansion-related factors after YAP1 manipulation and 3-MA treatment, with the aim of clarifying the relationship among YAP1, autophagy-associated signaling, and cumulus-cell molecular responses.

## 2. Materials and Methods

### 2.1. Ethics Statement

All procedures involving yak ovarian tissues and isolated cumulus cells were approved by the Ethics Committee of Gansu Agricultural University (protocol code GSAU-Eth-VMC-2022-009, approved in 2022). The ovaries used in this study were obtained as by-products from yaks slaughtered for commercial food production at a local abattoir in Linxia City, Gansu Province, China. No animals were slaughtered specifically for the purposes of this study.

### 2.2. Sample Collection

Yak ovaries were collected post mortem as abattoir by-products from commercially slaughtered adult female yaks at a local abattoir in Linxia City, Gansu Province, China. According to the available abattoir records, the donor animals were approximately 4–6 years old and showed no obvious signs of reproductive disease. Immediately after slaughter, the ovaries were rinsed with sterile physiological saline containing 1% penicillin–streptomycin and placed in an insulated container containing physiological saline maintained at approximately 30 °C. The samples were transported to the laboratory within 4 h after collection. Ovaries obtained from multiple animals within the same collection batch were pooled before cumulus–oocyte complex isolation and subsequent cell culture. The exact number and individual identities of donor animals contributing to each pooled preparation were not retained in the archived experimental records. Accordingly, pooled preparations were not treated as independent donor-level biological replicates.

### 2.3. Culture and Treatment of Yak Cumulus Cells

Ovarian tissues were rinsed three times with physiological saline containing 1% penicillin–streptomycin, prewarmed to 37 °C. Follicular fluid was selectively aspirated from well-developed follicles 2–8 mm in diameter using an 18-gauge needle, mixed with prewarmed oocyte collection medium (D-PBS + 5% FBS), and transferred to 50 mm culture dishes. Immature cumulus–oocyte complexes (COCs) were selected with an oocyte-picking needle under a stereomicroscope, transferred to oocyte collection medium prewarmed to 37 °C, washed three times with serum-free PBS, and cultured in maturation medium. After oocyte maturation, 1% hyaluronidase was added to digest the cumulus cells, and free cumulus cells were collected by gentle agitation. Following hyaluronidase digestion, the cumulus cells, together with the digestion mixture, were transferred to centrifuge tubes containing complete medium (DMEM/F-12 + 10% FBS + 100 U/mL penicillin + 100 μg/mL streptomycin) and centrifuged at 1500 rpm for 5 min to remove the supernatant. Digestion was terminated with complete medium, after which the cells were washed, gently pipetted into a single-cell suspension, seeded in 25 cm^2^ culture flasks, and maintained in primary culture at 37 °C in a 5% CO_2_ incubator. Cell identity was based on their origin from morphologically selected COCs and the established isolation procedure; no additional marker-based purity assay was performed in the present study. Passage-2 YCCs were seeded in six-well plates and transfected at approximately 80% confluence. For each well, cells received either 2.5 μg YAP1-overexpression plasmid or 100 pmol YAP1-targeting siRNA using 4 μL Lipo8000 Transfection Reagent (Beyotime Biotechnology, Shanghai, China). The corresponding empty vector (vector) and negative-control siRNA (si-NC) were used as controls. The siRNAs were obtained from GenePharma (Shanghai, China). The si-YAP1 sequences were sense 5′-GGCAAUGCGGAAUAUCAAUTT-3′ and antisense 5′-AUUGAUAUUCCGCAUUGCCTT-3′; the si-NC sequences were sense 5′-UUCUCCGAACGUGUCACGUTT-3′ and antisense 5′-ACGUGACACGUUCGGAGAATT-3′. Transfection treatment was performed for 24 h, and samples were collected 48 h after initiation of transfection for downstream analyses. Where indicated, cells were treated with 3-MA at a final concentration of 7.5 mM for 24 h. The four conditions in the YAP1-overexpression × 3-MA experiment were vector, 3-MA, YAP1-OE, and YAP1-OE + 3-MA.

### 2.4. RT-qPCR

Total RNA was extracted using TransZol reagent (TransGen, Beijing, China), and cDNA was synthesized using Evo M-MLV reagent (Accurate Biotechnology, Changsha, China). RT-qPCR was performed using SYBR Green Premix Pro Taq HS qPCR reagent (Accurate Biotechnology, Changsha, China) on a LightCycler 96 Real-Time PCR System (Roche, Basel, Switzerland). β-actin was used as the reference gene, and relative transcript abundance was calculated using the 2^−ΔΔCq^ method. β-actin was selected based on previous use in yak cumulus-cell qPCR studies [12]; a formal multi-reference-gene stability analysis was not performed prospectively in the present study. RT-qPCR analyses were based on three separately performed experimental runs (*n* = 3), each involving independently conducted cell culture/treatment, RNA extraction, reverse transcription, and qPCR. These experimental runs were used as the qPCR experimental units; no inferential statistics were based on within-run technical measurements. Archived LightCycler 96 project files showed run-specific thermocycling settings: initial denaturation at 95 °C for 30–600 s, followed by 40 or 45 cycles of 95 °C for 5 s, 56–58 °C for 30 s, and 72 °C for 15 s with real-time fluorescence acquisition; melting-curve analysis was subsequently performed from 65 to 97 °C. Prospective assay-specific standard-curve amplification-efficiency measurements were not retained, and unverified efficiency values were therefore not retrospectively introduced. Primer sequences and product sizes are shown in Table 1.

### 2.5. Western Blotting

The following proteins were detected in YCCs: the cumulus expansion-related proteins HAS2, PTX3, TNFAIP6, and PTGS2; and the autophagy-related proteins Beclin-1, P62, LC3B, and ATG5.

After the collected YCCs were washed, 150–250 μL of lysis buffer (RIPA:PMSF = 100:1) was added according to the number of cells in each well of the six-well plate, and total protein was extracted. Protein samples were mixed with 4× protein loading buffer at a 3:1 volume ratio, vortexed thoroughly, denatured in a dry heating block at 100 °C for 10 min to ensure complete protein dissociation and denaturation, and stored at −20 °C. The protein samples were separated by SDS-PAGE using 8% resolving gels and 5% stacking gels, after which the protein bands were transferred to PVDF membranes under constant-current conditions using a semi-dry transfer system. The transferred PVDF membranes were blocked in 5% skim-milk blocking solution for 2.5 h on a shaker at 70 rpm and then incubated with primary antibodies at 4 °C for 12–14 h. The primary antibodies included anti-YAP1 (1:4000, #13584-1-AP, Proteintech, Rosemont, IL, USA), anti-phospho-YAP1 (1:1000, #4911S, CST), anti-HAS2 (1:1000, #DF13702, Affinity Biosciences, Cincinnati, OH, USA), anti-PTX3 (1:1000, #13797-1-AP, Proteintech), anti-TNFAIP6 (1:1000, #AF5492, Affinity Biosciences), anti-PTGS2 (1:1000, #AF7003, Affinity Biosciences), anti-Beclin-1 (1:1500, #11306-1-AP, Proteintech), anti-P62 (1:1500, #18420-1-AP, Proteintech), anti-LC3B (1:1500, #14600-1-AP, Proteintech), anti-ATG5 (1:800, #BS4005R, Bioss, Woburn, MA, USA), and anti-β-actin (1:8000, #AF7018, Affinity Biosciences), which served as the internal control. The primary antibodies were diluted in PBST (0.1% Tween-20). After six washes with PBST (5 min each), the membranes were incubated with goat anti-rabbit IgG-HRP secondary antibody (1:8000, #M21002, AbMART, Shanghai, China) for 40 min at room temperature and then washed another six times with PBST (5 min each). Finally, signals were developed in the dark using ECL reagent (#E423-01, Vazyme Biotech Co., Ltd., Nanjing, China). Following exposure to X-ray film, the films were developed and fixed, and images of the protein bands were obtained by scanning. Western blot densitometry was based on three separately performed experimental runs (*n* = 3), each involving independently conducted cell culture/treatment, protein extraction, and immunoblotting. Band intensities were analyzed using ImageJ software (version 1.51j8).

### 2.6. Immunofluorescence Staining

Primary YCCs in good growth conditions were selected, digested, and seeded onto coverslips. When the cells had spread and reached approximately 60–70% confluence, they were washed three times with prewarmed PBS for 3 min each, fixed with 4% paraformaldehyde for 30 min at room temperature, and washed six times with PBS for 3 min each. The cells were permeabilized with 0.5% Triton X-100 solution (#IT9100, Solarbio, Beijing, China) for 15 min at room temperature and washed six times with PBS for 3 min each. They were then blocked with a 10% BSA solution for 30 min at room temperature and washed six times with PBS for 3 min each, followed by overnight incubation with primary antibodies at 4 °C. The primary antibodies included anti-YAP1 (1:400, #13584-1-AP, Proteintech), anti-PTGS2 (1:300, #AF7003, Affinity Biosciences), and a mouse anti-β-tubulin antibody used as a cytoskeletal marker, diluted in 5% BSA solution. After the primary antibodies were washed eight times with PBST for 3 min each, fluorescent secondary antibodies diluted in 5% BSA solution were added and incubated for 2 h at room temperature in the dark. The secondary antibodies included donkey anti-rabbit IgG (H+L) cross-adsorbed secondary antibody, Alexa Fluor 594-conjugated donkey anti-rabbit IgG (H+L) (1:800, #711-585-152, Jackson ImmunoResearch, West Grove, PA, USA), and donkey anti-mouse IgG (H+L) cross-adsorbed secondary antibody, Alexa Fluor 488-conjugated donkey anti-mouse IgG (H+L) (1:800, #715-545-150, Jackson ImmunoResearch, West Grove, PA, USA). Nuclei were stained with DAPI for 3–5 min, followed by six washes with PBST for 3 min each. The coverslips were mounted with antifade mounting medium, allowed to dry, and examined and photographed using a laser-scanning confocal fluorescence microscope.

### 2.7. Ad-mCherry-GFP-LC3B Assay

The adenovirus expressing the tandem mCherry-GFP-LC3B fusion protein was used to visualize LC3-related fluorescent puncta after infection of YCCs. When cell confluence exceeded 90%, the cells were digested and seeded onto coverslips. When the cells on the coverslips reached approximately 40% confluence, they were treated with Ad-mCherry-GFP-LC3B. After 24 h, the medium was replaced with fresh complete medium, and the cells were treated as described in Section 2.3. After treatment, the cells were washed three times with PBS, fixed with 4% paraformaldehyde at room temperature, washed with PBS to remove the fixative, stained with DAPI, washed with PBS, mounted with antifade mounting medium, and observed and photographed using a laser-scanning confocal fluorescence microscope. The reporter images were evaluated qualitatively; yellow and red-only puncta were not separately quantified, and lysosomal inhibitors were not included. Accordingly, this assay was not interpreted as a quantitative measurement of autophagic flux.

### 2.8. Statistical Analysis

**Figure 1:**
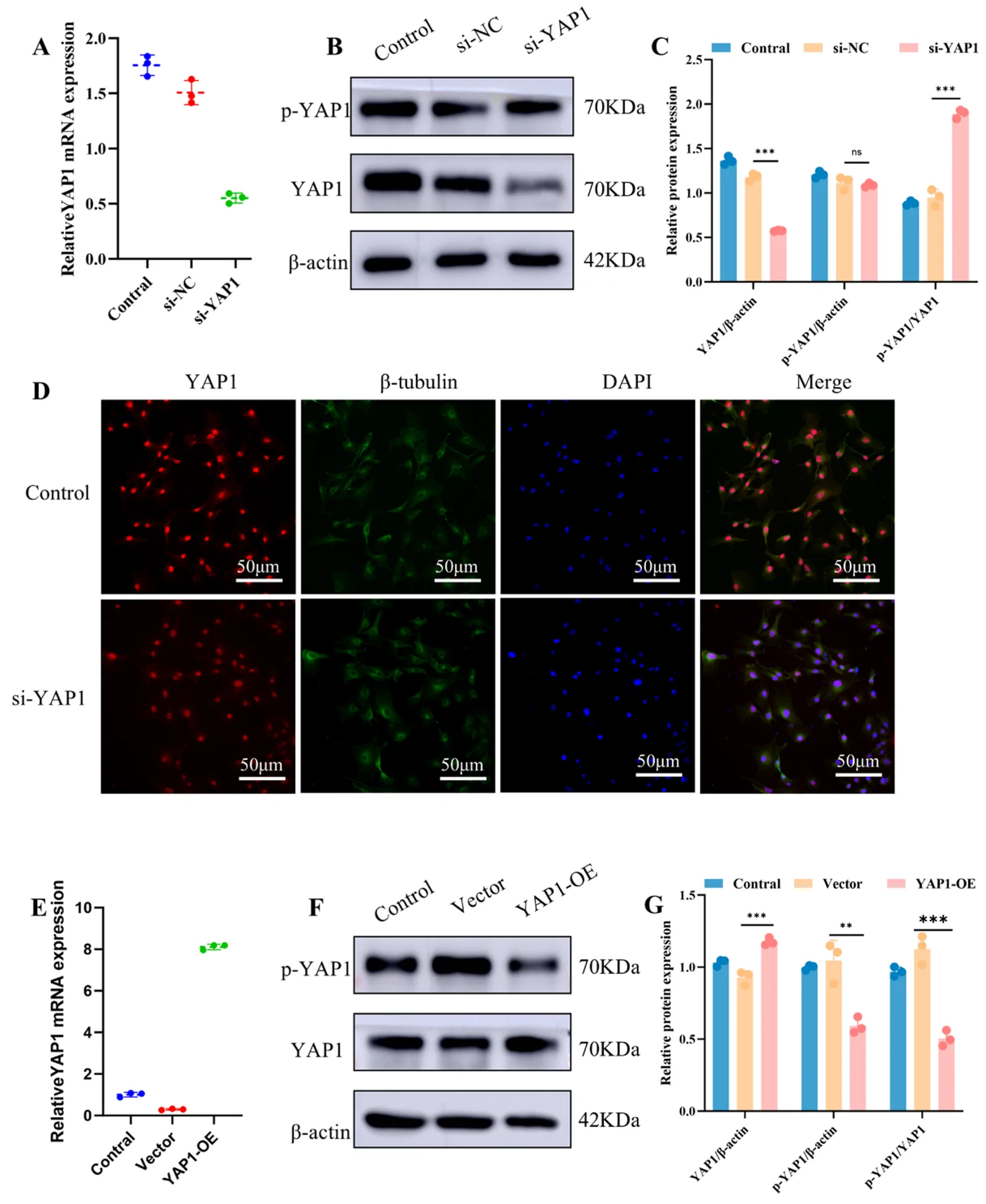

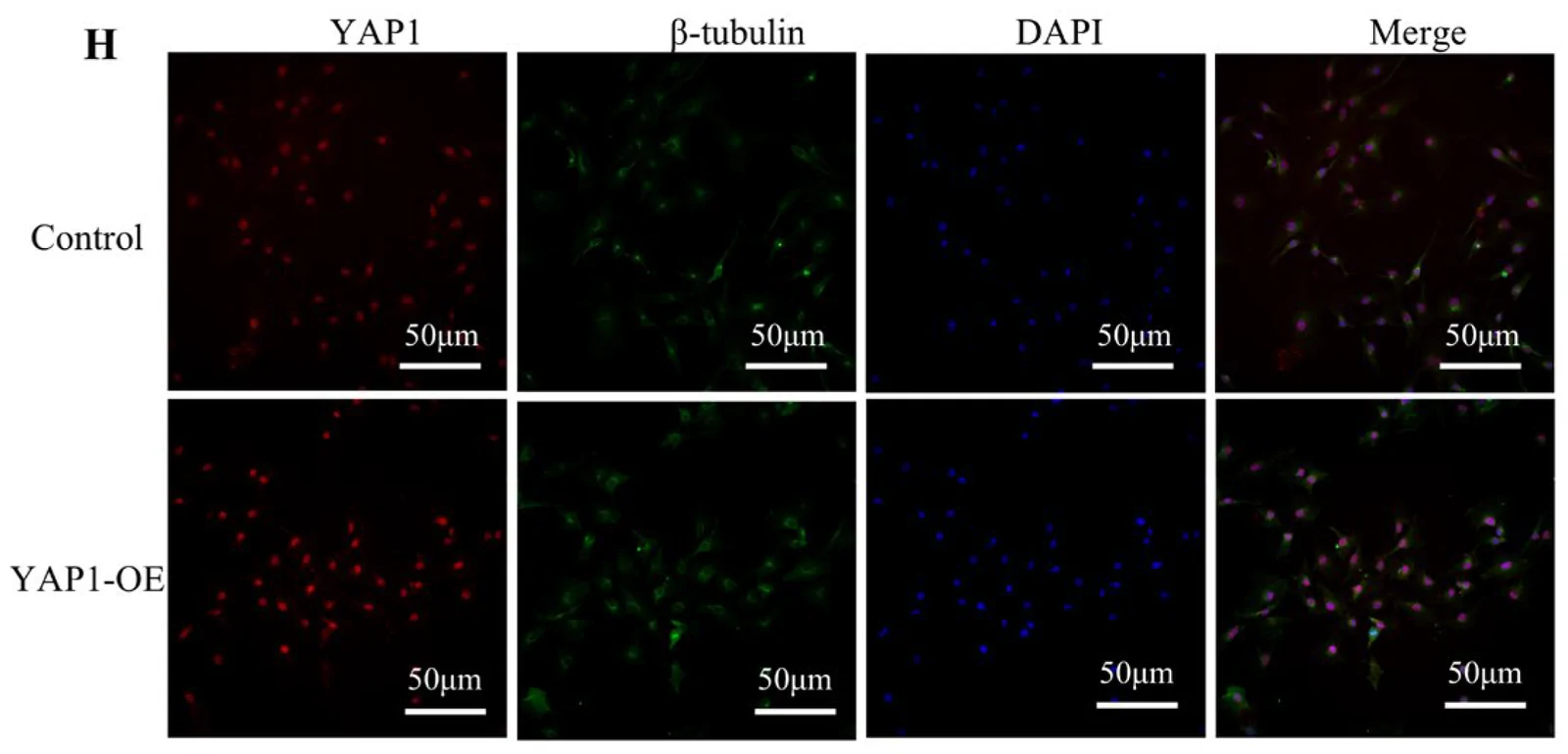
Validation of YAP1 knockdown and overexpression in yak cumulus cells. (**A**–**D**) Validation of YAP1 knockdown. (**A**) Relative YAP1 mRNA expression in the control, negative-control siRNA (si-NC), and YAP1-targeting siRNA (si-YAP1) groups, as determined by reverse transcription quantitative PCR (RT-qPCR). (**B**) Representative Western blots of phosphorylated YAP1 at Ser127 (p-YAP1), total YAP1, and β-actin. (**C**) Densitometric quantification of total YAP1 normalized to β-actin, p-YAP1 normalized to β-actin, and p-YAP1 normalized to total YAP1. (**D**) Representative immunofluorescence images showing YAP1 in red, β-tubulin in green, nuclei stained with DAPI in blue, and merged signals in the control and si-YAP1 groups. (**E**–**H**) Validation of YAP1 overexpression. (**E**) Relative YAP1 mRNA expression in the control, empty-vector control (vector), and YAP1-overexpression (YAP1-OE) groups, as determined by RT-qPCR. (**F**) Representative Western blots of p-YAP1, total YAP1, and β-actin. (**G**) Densitometric quantification of total YAP1 normalized to β-actin, p-YAP1 normalized to β-actin, and p-YAP1 normalized to total YAP1. (**H**) Representative immunofluorescence images showing YAP1 in red, β-tubulin in green, nuclei stained with DAPI in blue, and merged signals in the control and YAP1-OE groups. Scale bars = 50 μm. RT-qPCR panels display three separately performed experimental runs (*n* = 3), each involving independent cell culture/treatment, RNA extraction, and qPCR; these panels are presented descriptively and were not subjected to inferential statistical testing. Western blot densitometry was based on three separately performed experimental runs (*n* = 3) and was analyzed by one-way ANOVA followed by Tukey’s multiple-comparisons test. These runs were not interpreted as independent donor-level biological replicates. Graphical significance notation: ns, *p* ≥ 0.05; ** *p* < 0.01; *** *p* < 0.001. Adjusted *p*-values for the principal matched-control Western blot comparisons are reported in Section 3.1.

**Figure 2:**
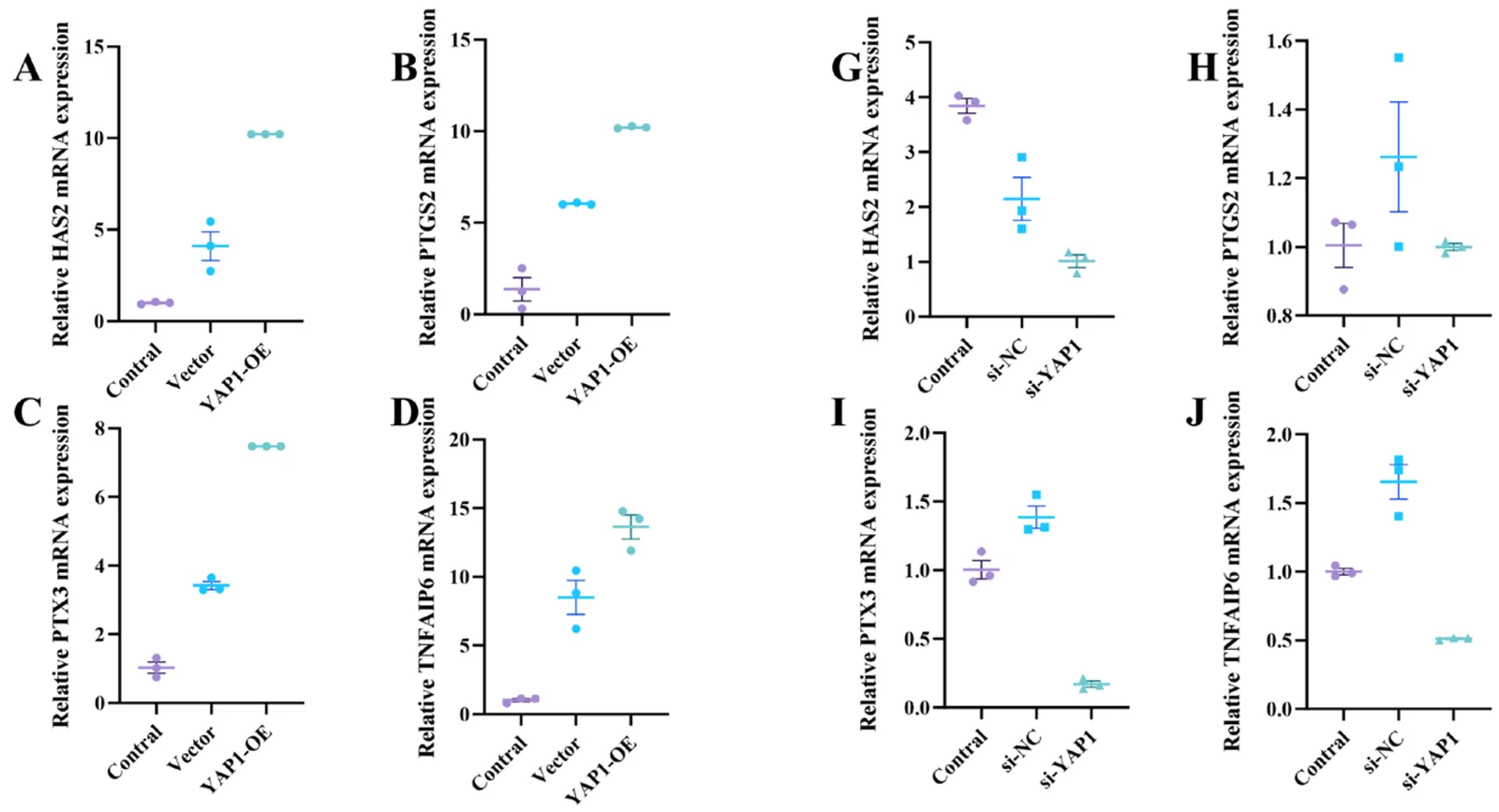

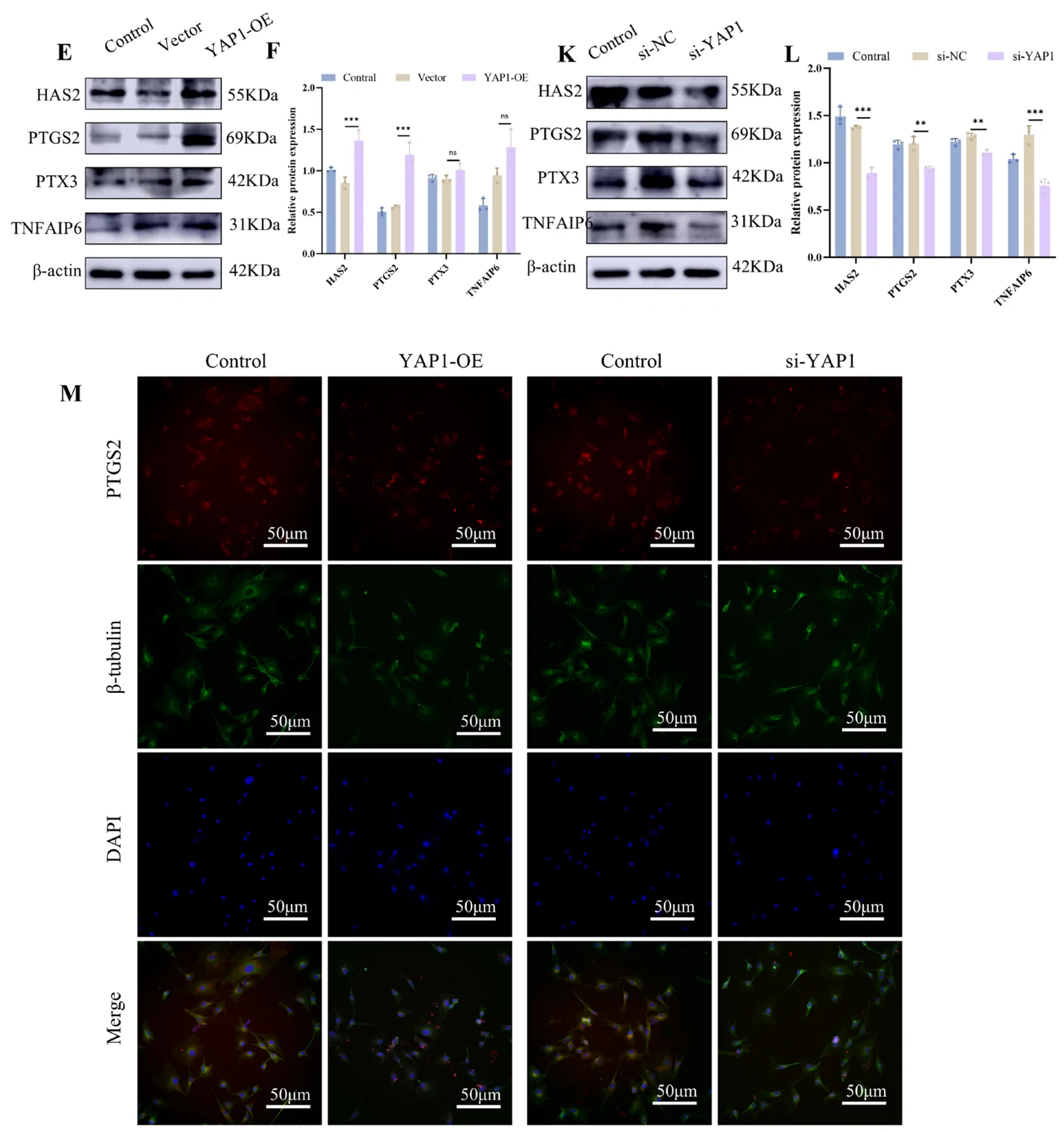
Effects of YAP1 overexpression and knockdown on the expression of cumulus expansion-related factors in yak cumulus cells. (**A**–**D**) Relative mRNA expression levels of HAS2, PTGS2, PTX3, and TNFAIP6, respectively, in the control, empty-vector control (vector), and YAP1-overexpression (YAP1-OE) groups, as determined by reverse transcription quantitative PCR (RT-qPCR). (**E**) Representative Western blots of HAS2, PTGS2, PTX3, TNFAIP6, and β-actin in the control, vector, and YAP1-OE groups. (**F**) Densitometric quantification of HAS2, PTGS2, PTX3, and TNFAIP6 protein abundance normalized to β-actin. (**G**–**J**) Relative mRNA expression levels of HAS2, PTGS2, PTX3, and TNFAIP6, respectively, in the control, negative-control siRNA (si-NC), and YAP1-targeting siRNA (si-YAP1) groups. (**K**) Representative Western blots of HAS2, PTGS2, PTX3, TNFAIP6, and β-actin in the control, si-NC, and si-YAP1 groups. (**L**) Densitometric quantification of HAS2, PTGS2, PTX3, and TNFAIP6 protein abundance normalized to β-actin. (**M**) Representative immunofluorescence images showing PTGS2/COX-2 in red, β-tubulin in green, nuclei stained with DAPI in blue, and merged signals in the corresponding control, YAP1-OE, and si-YAP1 groups. Scale bars = 50 μm. RT-qPCR panels display three separately performed experimental runs (*n* = 3), each involving independent cell culture/treatment, RNA extraction, and qPCR; these panels are presented descriptively and were not subjected to inferential statistical testing. Western blot densitometry was based on three separately performed experimental runs (*n* = 3) and was analyzed by one-way ANOVA followed by Tukey’s multiple-comparisons test. These runs were not interpreted as independent donor-level biological replicates. Graphical significance notation: ns, *p* ≥ 0.05; ** *p* < 0.01; *** *p* < 0.001. Adjusted *p*-values for the principal matched-control Western blot comparisons are reported in Section 3.2.

**Figure 3:**
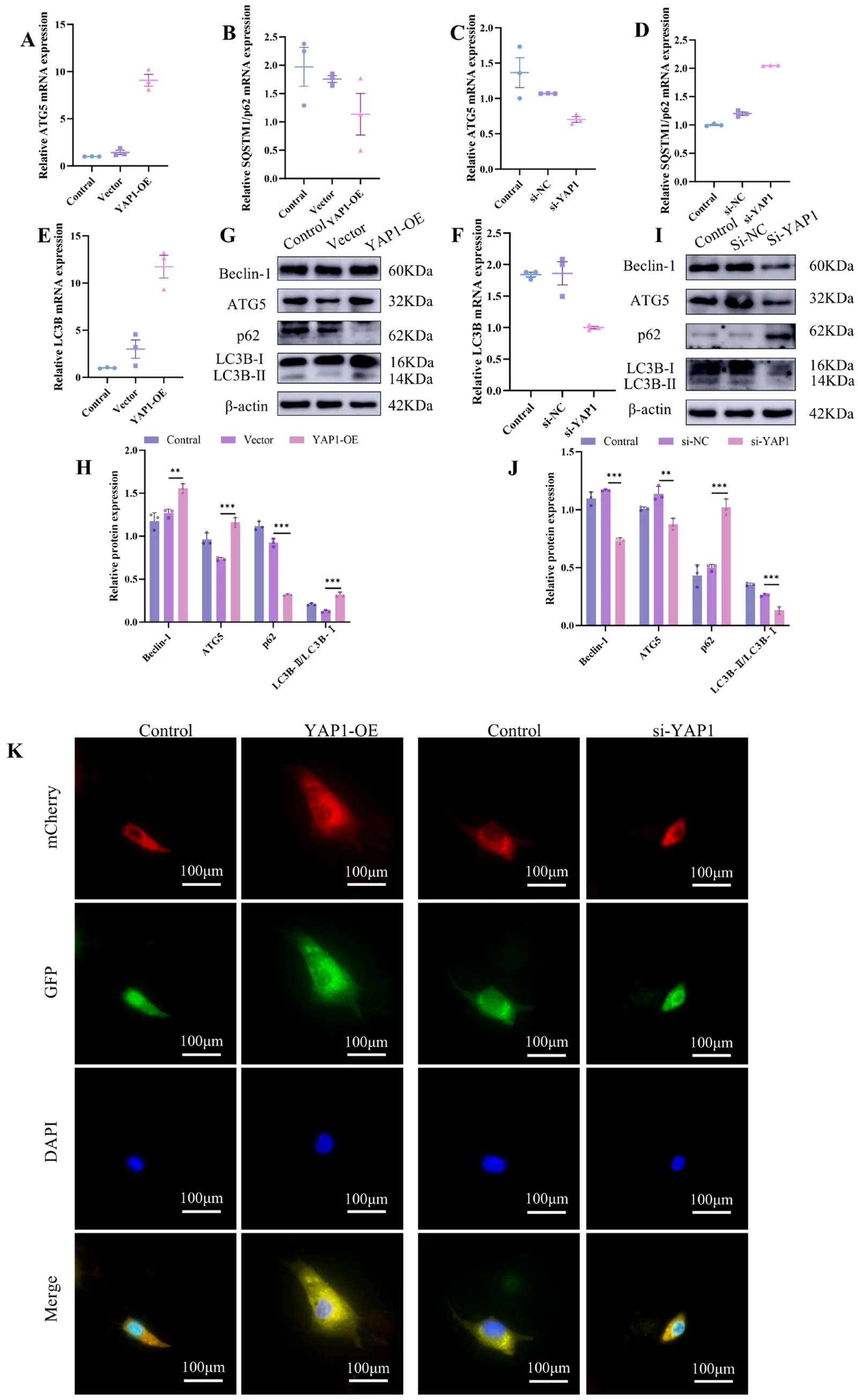
Effects of YAP1 overexpression and knockdown on autophagy-related markers in yak cumulus cells. (**A**,**B**,**E**) Relative mRNA expression levels of ATG5, SQSTM1/p62, and LC3B, respectively, in the control, empty-vector control (vector), and YAP1-overexpression (YAP1-OE) groups, as determined by reverse transcription quantitative PCR (RT-qPCR). (**C**,**D**,**F**) Relative mRNA expression levels of ATG5, SQSTM1/p62, and LC3B, respectively, in the control, negative-control siRNA (si-NC), and YAP1-targeting siRNA (si-YAP1) groups. (**G**) Representative Western blots of Beclin-1, ATG5, p62, LC3B-I, LC3B-II, and β-actin in the control, vector, and YAP1-OE groups. (**H**) Densitometric quantification of Beclin-1, ATG5, and p62 normalized to β-actin and of the LC3B-II/LC3B-I ratio in the YAP1-overexpression experiment. (**I**) Representative Western blots of Beclin-1, ATG5, p62, LC3B-I, LC3B-II, and β-actin in the control, si-NC, and si-YAP1 groups. (**J**) Densitometric quantification of Beclin-1, ATG5, and p62 normalized to β-actin and of the LC3B-II/LC3B-I ratio in the YAP1-knockdown experiment. (**K**) Representative fluorescence images of yak cumulus cells expressing the tandem mCherry-GFP-LC3B reporter in the control, YAP1-OE, and si-YAP1 groups. mCherry fluorescence is shown in red, GFP fluorescence in green, and nuclei stained with DAPI in blue, and the corresponding merged channels are shown in the bottom row. Scale bars = 100 μm. RT-qPCR panels display three separately performed experimental runs (*n* = 3), each involving independent cell culture/treatment, RNA extraction, and qPCR; these panels are presented descriptively and were not subjected to inferential statistical testing. Western blot densitometry was based on three separately performed experimental runs (*n* = 3) and was analyzed by one-way ANOVA followed by Tukey’s multiple-comparisons test. These runs were not interpreted as independent donor-level biological replicates. LC3B-I and LC3B-II are displayed separately in representative blots, whereas quantitative LC3B protein results are expressed as the LC3B-II/LC3B-I ratio. Graphical significance notation: ns, *p* ≥ 0.05; ** *p* < 0.01; *** *p* < 0.001. Adjusted *p*-values are reported in Section 3.3.

**Figure 4:**
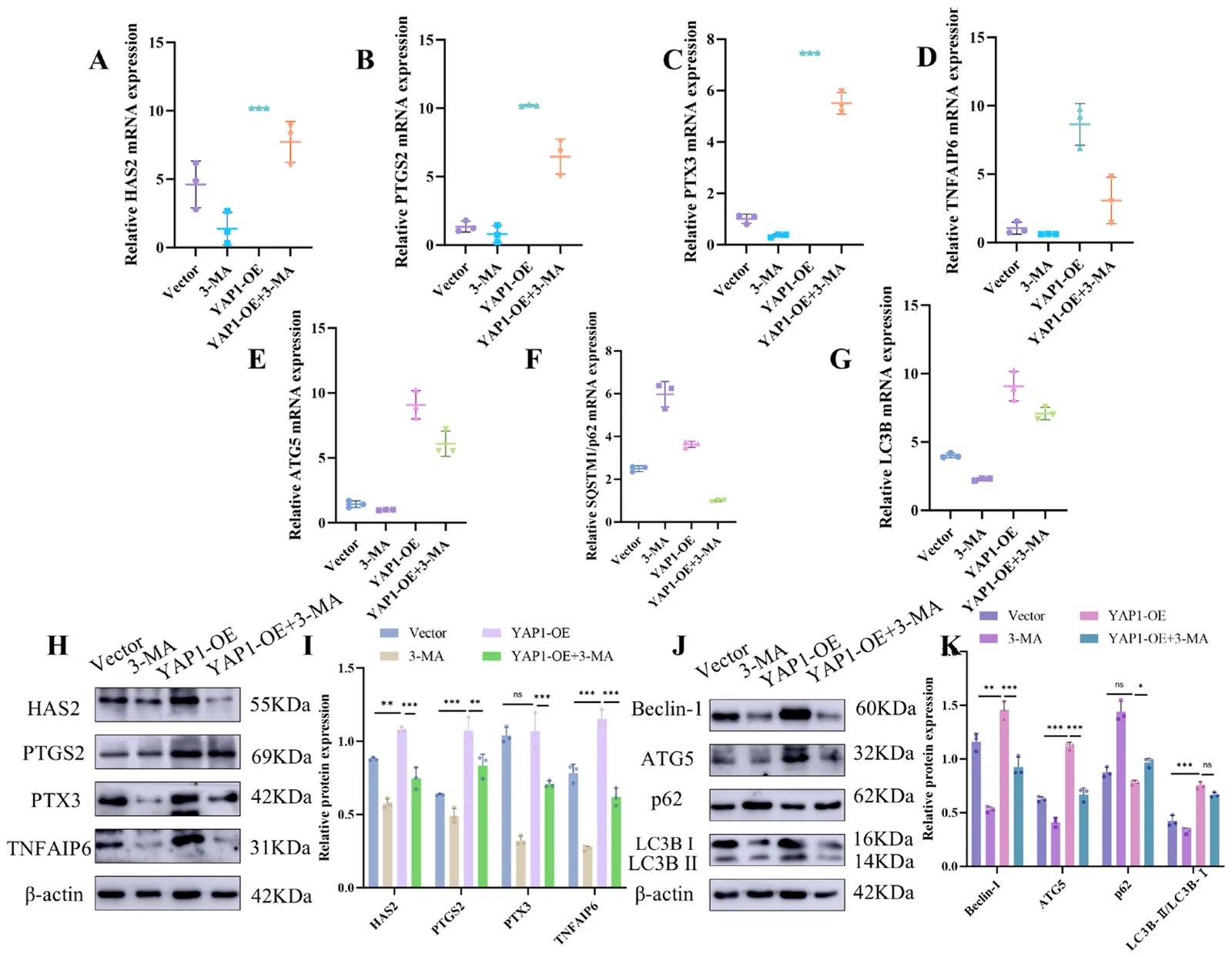
3-MA co-treatment attenuates several YAP1-overexpression-associated molecular changes. The four treatment conditions were vector, 3-MA, YAP1-OE, and YAP1-OE + 3-MA. (**A**–**D**) Relative mRNA expression levels of HAS2, PTGS2, PTX3, and TNFAIP6, respectively, as determined by RT-qPCR. (**E**–**G**) Relative mRNA expression levels of ATG5, SQSTM1/p62, and LC3B, respectively. (**H**) Representative Western blots of HAS2, PTGS2, PTX3, TNFAIP6, and β-actin. (**I**) Densitometric quantification of HAS2, PTGS2, PTX3, and TNFAIP6 protein abundance normalized to β-actin. (**J**) Representative Western blots of Beclin-1, ATG5, p62, LC3B-I, LC3B-II, and β-actin. (**K**) Densitometric quantification of Beclin-1, ATG5, and p62 normalized to β-actin and of the LC3B-II/LC3B-I ratio. RT-qPCR panels display three separately performed experimental runs (*n* = 3), each involving independent cell culture/treatment, RNA extraction, and qPCR; these panels are presented descriptively and were not subjected to inferential statistical testing. Western blot densitometry was based on three separately performed experimental runs (*n* = 3); these runs were not interpreted as independent donor-level biological replicates. Western blot data were analyzed by two-way ANOVA with YAP1 overexpression and 3-MA treatment as factors, followed by Tukey-adjusted pairwise comparisons. LC3B-I and LC3B-II are displayed separately in representative blots, whereas the corresponding quantitative LC3B result is expressed as the LC3B-II/LC3B-I ratio. Graphical significance notation: ns, *p* ≥ 0.05; * *p* < 0.05; ** *p* < 0.01; *** *p* < 0.001. Adjusted *p*-values for the principal comparisons are reported in Section 3.4.

Statistical analyses of Western blot densitometry were performed using SPSS 21.0 (IBM Corp., Armonk, NY, USA), with graphical visualization in GraphPad Prism 8 (GraphPad Software, Inc., San Diego, CA, USA). Western blot densitometry was based on three separately performed experimental runs (*n* = 3), each involving independently conducted cell culture/treatment, protein extraction, and immunoblotting. These experimental runs were used as the experimental units for Western blot statistical analysis. Because ovaries from multiple animals were pooled and individual donor identities were not retained, these runs were not interpreted as independent donor-level biological replicates, and donor-to-donor variability could not be evaluated. Model residuals were assessed using the Shapiro–Wilk test and homogeneity of variance using Levene’s test; no clear violations were detected, although the small sample size limits the power of these diagnostic tests. For Figure 1, Figure 2 and Figure 3, Western blot data were analyzed by one-way ANOVA followed by Tukey’s multiple-comparisons test. For the four-group YAP1-overexpression × 3-MA experiment in Figure 4, two-way ANOVA was used to evaluate the main effects of YAP1 overexpression and 3-MA and their interaction, followed by Tukey-adjusted pairwise comparisons. RT-qPCR analyses were based on three separately performed experimental runs (*n* = 3), each involving independently conducted cell culture/treatment, RNA extraction, reverse transcription, and qPCR. These runs were used as the qPCR experimental units and were presented descriptively; no inferential statistics were based on within-run technical measurements. Representative immunofluorescence and mCherry-GFP-LC3B images were evaluated qualitatively. Western blot data are expressed as mean ± SEM. *p* < 0.05 was considered statistically significant.

## 3. Results

### 3.1. Validation of YAP Overexpression and Knockdown in Yak Cumulus Cells

YAP1 protein measurements were first examined to validate the gain- and loss-of-function models. Relative to the empty-vector control, YAP1 overexpression significantly increased total YAP1/β-actin (Tukey-adjusted *p* = 2.93 × 10^−4^), while p-YAP1/β-actin and the p-YAP1/total YAP1 ratio were reduced (*p* = 0.00178 and *p* = 7.48 × 10^−5^, respectively). Relative to si-NC, si-YAP1 significantly reduced total YAP1/β-actin (*p* = 2.41 × 10^−6^) and increased the p-YAP1/total YAP1 ratio (*p* = 4.15 × 10^−6^), whereas p-YAP1/β-actin was not significantly altered (*p* = 0.780). RT-qPCR experimental runs showed corresponding directional changes in YAP1 transcript abundance and were presented descriptively. Representative immunofluorescence images qualitatively showed increased YAP1 fluorescence following overexpression and decreased fluorescence following knockdown; nuclear/cytoplasmic YAP1 distribution was not quantitatively assessed. Together, these data confirm effective manipulation of YAP1 abundance in YCCs (Figure 1).

### 3.2. YAP1 Manipulation Is Associated with Changes in Cumulus Expansion-Related Factors

After validating YAP1 manipulation, we examined HAS2, PTGS2, PTX3, and TNFAIP6. Relative to the vector control, YAP1 overexpression significantly increased HAS2 and PTGS2 protein abundance (Tukey-adjusted *p* = 5.26 × 10^−4^ and *p* = 2.59 × 10^−4^, respectively), whereas PTX3 and TNFAIP6 showed upward trends that did not reach statistical significance (*p* = 0.140 and *p* = 0.0539, respectively). In contrast, YAP1 knockdown significantly reduced HAS2, PTGS2, PTX3, and TNFAIP6 protein abundance relative to si-NC (*p* = 2.65 × 10^−4^, *p* = 0.00191, *p* = 0.00158, and *p* = 2.60 × 10^−4^, respectively). RT-qPCR experimental runs showed corresponding directional changes and were interpreted descriptively. Representative PTGS2 immunofluorescence images were qualitatively consistent with the Western blot pattern. These data indicate that YAP1 manipulation is associated with changes in selected molecular factors related to cumulus expansion, with the clearest overexpression-associated protein increases observed for HAS2 and PTGS2 (Figure 2).

### 3.3. YAP Manipulation Is Associated with Changes in Autophagy-Related Markers

To assess the relationship between YAP1 and autophagy-associated processes, we evaluated Beclin-1, ATG5, p62, and LC3B. Relative to the vector control, YAP1 overexpression significantly increased Beclin-1 and ATG5 abundance and the LC3B-II/LC3B-I ratio (Tukey-adjusted *p* = 0.00469, *p* = 1.57 × 10^−4^, and *p* = 4.56 × 10^−5^, respectively) and decreased p62 abundance (*p* = 4.19 × 10^−6^). Relative to si-NC, YAP1 knockdown significantly decreased Beclin-1, ATG5, and the LC3B-II/LC3B-I ratio (*p* = 1.45 × 10^−5^, *p* = 0.00133, and *p* = 3.96 × 10^−4^, respectively) and increased p62 abundance (*p* = 2.17 × 10^−4^). RT-qPCR experimental runs showed corresponding directional changes and were interpreted descriptively. Representative mCherry-GFP-LC3B images showed qualitative treatment-associated differences in LC3-related punctate signals (Figure 3). These findings demonstrate YAP1-associated changes in autophagy-related molecular markers.

### 3.4. 3-MA Co-Treatment Attenuates Several YAP1-Overexpression-Associated Molecular Changes

We next examined whether 3-MA modified the YAP1-overexpression-associated autophagy-related protein pattern. Relative to vector, YAP1 overexpression significantly increased Beclin-1 and ATG5 abundance and the LC3B-II/LC3B-I ratio (Tukey-adjusted *p* = 0.00528, *p* = 2.42 × 10^−6^, and *p* = 1.20 × 10^−5^, respectively), whereas the decrease in p62 did not reach statistical significance (*p* = 0.274). Compared with YAP1 overexpression alone, 3-MA co-treatment significantly reduced Beclin-1 and ATG5 abundance (*p* = 1.07 × 10^−4^ and *p* = 4.97 × 10^−6^, respectively) and increased p62 abundance (*p* = 0.0196). The LC3B-II/LC3B-I ratio was numerically lower after co-treatment, but the pairwise difference did not reach statistical significance (*p* = 0.0526). Thus, 3-MA attenuated several, but not all, autophagy-associated protein changes observed after YAP1 overexpression.

For cumulus expansion-related proteins, YAP1 overexpression significantly increased HAS2, PTGS2, and TNFAIP6 relative to vector (Tukey-adjusted *p* = 0.00170, *p* = 1.40 × 10^−4^, and *p* = 1.15 × 10^−4^, respectively), whereas PTX3 was not significantly changed (*p* = 0.948). Compared with YAP1 overexpression alone, 3-MA co-treatment significantly reduced HAS2, PTGS2, PTX3, and TNFAIP6 protein abundance (*p* = 5.01 × 10^−5^, *p* = 0.00749, *p* = 9.64 × 10^−4^, and *p* = 7.66 × 10^−6^, respectively). YAP1 × 3-MA interactions were significant for PTX3, ATG5, and p62 (*p* = 0.00226, *p* = 0.00116, and *p* = 4.71 × 10^−4^, respectively) but not for the other markers examined. These findings show that several YAP1-overexpression-associated molecular changes are sensitive to 3-MA treatment, without establishing a uniform or pathway-specific causal mechanism (Figure 4).

## 4. Discussion

Cumulus expansion is an important event in oocyte maturation and the acquisition of developmental competence. The present study systematically examined the relationship between YAP1, autophagy-related markers, and the expression of expansion-related factors in yak cumulus cells. The results showed that YAP1 overexpression was associated with increased expression of several cumulus expansion-related proteins and changes in autophagy-related markers, whereas YAP1 knockdown produced an overall opposite pattern; treatment with 3-MA further attenuated several molecular changes associated with YAP1 overexpression. These findings suggest that YAP1 is associated with the regulation of cumulus expansion-related molecular programs in yak cumulus cells and that autophagy-associated signaling may contribute to these responses. However, direct functional cumulus expansion and autophagic flux were not established in the present study.

As a core transcriptional co-activator of the Hippo signaling pathway, the biological effects of YAP depend mainly on its phosphorylation status and subcellular localization. When the Hippo pathway is activated, LATS1/2 mediates YAP phosphorylation and retains it in the cytoplasm; when YAP is dephosphorylated and enters the nucleus, it can bind to TEAD family transcription factors and regulate genes associated with cell proliferation, survival, metabolism, and the extracellular matrix. Previous studies have shown that the timely expression and activation of YAP in granulosa cells are important for follicular growth and granulosa-cell differentiation [15,16]. In bovine preovulatory granulosa cells, disruption of the YAP-TEAD interaction reduces the expression of the ovulation-related genes EGFR, EREG, EGR1, and TNFAIP6 and markedly affects GnRH-induced ovulation [19]; studies in bovine cumulus cells have also shown that EGFR activation increases the expression of the canonical YAP/TAZ-TEAD target gene CTGF, indicating a functional interaction between EGF-like signaling and YAP/TAZ [20]. In addition, FHL2 deficiency impairs follicular development and fertility by weakening EGF/EGFR/YAP signaling [21]. In the present study, YAP1 overexpression was associated with changes in cumulus expansion-related factors, with the clearest protein-level increases observed for HAS2 and PTGS2, suggesting that YAP1 may participate in pro-ovulatory molecular signaling in yak cumulus cells. A recent yak study further showed that activation of YAP1 and TAZ promotes oocyte maturation and regulates the expression of cumulus expansion factors and oocyte-secreted factors [1]. Thus, the present findings are generally consistent with previous evidence implicating Hippo-YAP signaling in ovarian somatic-cell function and oocyte maturation. However, nuclear/cytoplasmic YAP1 distribution and canonical YAP1-TEAD target genes were not quantitatively evaluated in the present study; therefore, direct activation of YAP1-TEAD transcription cannot be concluded from the current data.

During expansion, HAS2 is responsible for the synthesis of the hyaluronan backbone; TSG-6, encoded by TNFAIP6, participates in hyaluronan cross-linking; PTX3 acts as an oligomeric matrix protein that maintains the stability of the cumulus matrix; and PTGS2 participates in amplification of ovulatory signaling through prostaglandin synthesis [22]. Deficiency of PTX3 or TNFAIP6 disrupts cumulus matrix formation and reduces female fertility, indicating that these molecules are not only markers of cumulus expansion but also key effector molecules required to maintain matrix structure and ovulatory function [23,24]. The present study showed coordinated but marker-dependent changes in HAS2, PTGS2, PTX3, and TNFAIP6 following YAP1 manipulation. YAP1 overexpression produced the clearest protein-level increases in HAS2 and PTGS2, whereas YAP1 knockdown significantly reduced all four proteins, suggesting that YAP1 may affect multiple molecular processes associated with hyaluronan synthesis, matrix organization, and ovulatory signaling. Notably, HAS2 and PTGS2 showed the clearest protein-level responses to YAP1 overexpression, whereas the responses of PTX3 and TNFAIP6 were more modest. This difference may reflect distinct regulatory inputs or temporal responses among these genes: HAS2 and PTGS2 are more closely associated with initiation and signal amplification during cumulus expansion, whereas PTX3 and TNFAIP6 are also regulated by oocyte-secreted factors, EGF-like signaling, and the pre-existing state of the matrix. Previous studies have shown that oocyte-secreted factors can reset the responsiveness of cumulus cells to FSH and the maturation cascade and alter the expression of genes including HAS2, PTGS2, PTX3, and TNFAIP6 [4]. Therefore, the non-proportional changes among expansion-related factors observed in this study may reflect differential regulation within the cumulus expansion-related molecular network, although experimental variability cannot be excluded. Because direct binding of YAP1/TEAD to the regulatory regions of these genes was not examined in the present study, they are more appropriately considered YAP1-responsive molecules rather than established direct transcriptional targets of YAP1.

Autophagy is a dynamic degradative process that maintains cellular homeostasis by removing damaged mitochondria and abnormal proteins, recycling amino acids and lipids, and preserving endoplasmic reticulum and lysosomal function [25,26]. During maturation, cumulus cells must synthesize large amounts of hyaluronan and matrix proteins while maintaining hormone secretion and the delivery of metabolic substrates to the oocyte; therefore, they have a high demand for organelle quality control and energy redistribution. The hypoxic and metabolically variable environment associated with yak ovarian physiology may increase the importance of adaptive cellular processes such as autophagy in maintaining cumulus-cell homeostasis. Oxidative signals may also contribute to the regulation of autophagy in ovarian somatic cells. In mouse granulosa cells, AQP8-mediated hydrogen peroxide transport was associated with LC3-related autophagy and follicular atresia, whereas Aqp8 deficiency reduced autophagosome abundance [27]. These results provide additional evidence that redox status and autophagy are functionally linked in granulosa cells. Nevertheless, the follicular atresia model used in that study differs from the cultured yak cumulus-cell model examined here. Previous studies have shown that HIF-1α can improve yak cumulus expansion, oocyte maturation, and early embryonic development by regulating autophagy, whereas 3-MA weakens these effects [2]; EGF regulates autophagy in yak cumulus cells in a marked concentration-dependent manner [12]; and exosomes derived from yak follicular fluid can be taken up by cumulus cells and promote the expression of estrogen metabolism-related molecules through activation of autophagy [11]. A recent study in bovine oocytes further showed that Mogroside III enhances autophagy, hormone secretion, and cumulus expansion in cumulus cells and improves oocyte maturation and subsequent embryonic development [14]; an appropriate level of rapamycin can also improve the developmental competence of yak oocytes by enhancing autophagy [13]. These studies indicate that moderate autophagy can support metabolism, secretion, and matrix remodeling in cumulus cells. A protective role of autophagy has also been demonstrated in bovine cumulus–oocyte complexes under heat-stress conditions. Inhibition of autophagy with 3-MA reduced the LC3B-II/LC3B-I ratio in cumulus cells and further impaired cleavage and blastocyst development following heat-shock treatment during oocyte maturation [28]. This finding supports the view that autophagy can function as an adaptive mechanism during bovine oocyte maturation. However, because this effect was demonstrated under heat stress, it does not imply that increasing autophagy is uniformly beneficial under all culture conditions. In the present study, YAP1 overexpression increased Beclin-1 and ATG5 abundance and the LC3B-II/LC3B-I ratio while decreasing p62 abundance, whereas YAP1 knockdown produced the opposite overall pattern. The tandem mCherry-GFP-LC3B reporter was originally developed to distinguish autophagic structures at different maturation stages because GFP fluorescence is more readily quenched following lysosomal acidification, whereas mCherry fluorescence is relatively retained [29]. However, representative reporter images and static changes in LC3B, p62, Beclin-1, and ATG5 do not by themselves establish altered autophagic flux. Because yellow- and red-only puncta were not separately quantified and lysosomal inhibitors were not included, the present findings are more appropriately interpreted as YAP1-associated changes in autophagy-related molecular markers rather than definitive evidence of altered autophagic flux [26,29].

To determine whether autophagy-associated signaling participates in the regulation of cumulus expansion-related factors by YAP1, 3-MA was applied in the context of YAP1 overexpression [30]. Compared with YAP1 overexpression alone, 3-MA co-treatment significantly decreased Beclin-1 and ATG5 abundance and increased p62, whereas the LC3B-II/LC3B-I ratio was numerically lower but did not reach statistical significance. 3-MA co-treatment also significantly reduced HAS2, PTGS2, PTX3, and TNFAIP6 protein abundance relative to YAP1 overexpression alone. These findings indicate that several YAP1-overexpression-associated molecular changes are sensitive to 3-MA treatment and are consistent with partial involvement of autophagy-associated signaling. However, because 3-MA is not specific to autophagy and can affect class I and class III PI3K signaling in a time- and condition-dependent manner [30], the present pharmacological data do not establish that autophagy specifically mediates the effects of YAP1. Contributions from PI3K-AKT-mTOR signaling or other autophagy-independent effects of 3-MA therefore cannot be excluded. Previous research has shown that autophagy promotes ovarian granulosa-cell differentiation through degradation of WT1 and affects FSHR expression and hormone secretion [31]. Therefore, YAP1-associated changes in autophagy-related processes may influence the responsiveness of cumulus cells to FSH, EGF, and ovulatory signals through mechanisms that remain to be defined. Cross-talk between Hippo-YAP signaling and autophagy has also been observed in ovarian granulosa cells. In chicken granulosa cells, the miR-34a-5p/LEF1 axis promoted autophagy and apoptosis through modulation of Hippo-YAP signaling [32]. This study provides cross-species evidence that Hippo-YAP signaling can interact with the autophagic machinery in ovarian somatic cells. However, because it focused on follicular atresia and apoptosis in an avian model, it should be regarded as supportive evidence for pathway interaction rather than direct confirmation of the mechanism proposed in yak cumulus cells. The incomplete correspondence among YAP1-, 3-MA-, and marker-specific responses further suggests that YAP1 may influence cumulus-cell molecular programs through multiple pathways, potentially including TEAD-dependent transcription, EGFR-ERK, PI3K-AKT, AMPK-mTOR, and cytoskeletal signaling. Thus, autophagy-associated signaling may represent one component of the molecular response associated with YAP1 manipulation rather than the sole mechanism involved. Genetic inhibition of ATG5, BECN1, or other essential autophagy-related genes will be required to distinguish autophagy-dependent effects from other pharmacological actions of 3-MA.

The present study suggests a potential relationship among YAP1 signaling, autophagy-related molecular changes, and cumulus expansion-related molecular programs in yak cumulus cells. YAP1 overexpression was associated with increases in several cumulus expansion-related proteins and changes in autophagy-related markers, whereas YAP1 knockdown produced an overall opposite pattern. In addition, 3-MA co-treatment attenuated several molecular changes associated with YAP1 overexpression, supporting the possibility that 3-MA-sensitive, autophagy-associated signaling contributes to part of the YAP1-associated molecular response. One possible working model is that YAP1 may influence cumulus expansion-related molecular programs through YAP1/TEAD-dependent transcription and interactions with EGF-like signaling, while autophagy-related processes may contribute to cellular metabolic and proteostatic adaptation [15,20,33]. These mechanisms could in turn influence molecular processes associated with extracellular matrix remodeling. However, the present study did not directly assess YAP1/TEAD transcriptional activity, autophagic flux, or functional matrix formation; therefore, this model should be regarded as a hypothesis requiring further experimental validation. Such interactions may also be influenced by hypoxia, oxidative stress, cell junctions, and changes in matrix mechanics. Future combined interventions targeting YAP/TEAD and autophagy-related genes in intact yak cumulus–oocyte complexes, together with analyses linking molecular changes to cumulus expansion area, hyaluronan content, first polar body extrusion rate, fertilization rate, and blastocyst rate, will help further establish the physiological significance of this proposed regulatory relationship.

Several limitations of the present study should be considered. First, yak ovaries collected within the same batch were pooled before cumulus-cell isolation, and the exact number and individual identities of contributing donor animals were not retained; therefore, donor-to-donor variability could not be evaluated. Second, RT-qPCR analyses were based on three separately performed experimental runs (*n* = 3), each involving independently conducted cell culture/treatment, RNA extraction, reverse transcription, and qPCR; however, the number of runs was small, so the RT-qPCR results were interpreted descriptively. Third, cumulus-cell identity was based on their isolation from selected cumulus–oocyte complexes, and additional marker-based purity characterization was not performed. Fourth, the study focused on molecular markers and did not directly assess cumulus expansion, hyaluronan production, oocyte maturation, fertilization, or embryo development. In addition, YAP1 nuclear/cytoplasmic distribution and canonical YAP1-TEAD target genes were not quantitatively evaluated. Finally, autophagic flux was not established using quantitative tandem-reporter analysis or lysosomal blockade, and 3-MA was used without complementary genetic inhibition of key autophagy-related genes. These limitations should be addressed in future studies using independently derived donor preparations, intact yak cumulus–oocyte complexes, quantitative autophagic-flux assays, and genetic manipulation of autophagy-related pathways.

## 5. Conclusions

Under the experimental conditions used, YAP1 manipulation was associated with coordinated changes in cumulus expansion-related and autophagy-related molecular markers in yak cumulus cells. YAP1 overexpression showed the clearest protein-level increases in HAS2 and PTGS2, whereas YAP1 knockdown reduced all four expansion-related proteins examined. 3-MA co-treatment attenuated several YAP1-overexpression-associated molecular changes, supporting the possibility that 3-MA-sensitive, autophagy-associated signaling contributes to part of this response. However, the present data do not establish functional cumulus expansion, altered autophagic flux, or pathway-specific causality. Direct functional assays in intact cumulus–oocyte complexes, quantitative autophagic-flux measurements, and pathway-specific genetic validation are required to establish the proposed mechanism.

## Figures and Tables

**Table 1:** RT-qPCR primers.

Gene	Primer Sequence	Product Size (bp)
*β-actin*	F: CGTCCGTGACATCAAGGAGAAGC R: GGAACCGCTCATTGCCGATGG	141
YAP1	F: CTGACGATGTGCCTCTGCC R: GAGGTGGTCTTGTTCTTGTGGTT	268
*HAS2*	F: ACAGGCATCTAACGAACCGAG R: AGTAGGACTTGCTCCAGCGG	135
*PTX3*	F: GCTATCGGTCCATAATGCTTG R: CCACCGAGTCACCATTTACC	150
*TNFAIP6*	F: AGCAGTTAGAGGCAGCCAGAAA R: AACACACCACCACACTCCTTTG	211
PTGS2	F: CGTTTTCTCGTGAAGCCCTAT R: CAGTACTCGGGAGAGCATATAGG	230
SQSTM1/p62	F: ATCAGCCTCTGGTCCATC R: TTCTCTTGCCTCCGTGTT	128
*LC3B*	F: TGTTAGGTCAGGCAGTCA R: GTAGTAGGAAGCACTCGTTA	150
*ATG5*	F: ATCAATCGGAAACTCATG R: AGATGTTCACTCAGCCAC	272

## Data Availability

The datasets generated during and/or analyzed during the current study are available from the corresponding author upon reasonable request.
